# Enhancing Clinical Medical Education Through Hand Gesture Analogy Teaching: A Narrative Review

**DOI:** 10.7759/cureus.76619

**Published:** 2024-12-30

**Authors:** Guangbin Chen, Mingliang Xu, Qi Wang, Chunyan Xu, Yiwen Wang, Tingting Wu, Yifen Ma, Linglong Peng, Xuelei Ji

**Affiliations:** 1 Department of Hepatobiliary Surgery, The Second People's Hospital of Wuhu, Wuhu Hospital Affiliated to East China Normal University, Wuhu, CHN; 2 Department of Teaching and Research, The Second People's Hospital of Wuhu, Wuhu Hospital Affiliated to East China Normal University, Wuhu, CHN

**Keywords:** anatomical education, educational innovation, hand-foot teaching, hand gesture teaching, medical education, medical training technology, non-verbal communication, pedagogical methods

## Abstract

This narrative review assesses the effectiveness of hand gesture analogy teaching in clinical medical education, addressing the growing demand for innovative pedagogical strategies. Through a comprehensive analysis of existing literature, it evaluates the theoretical foundations, implementation strategies, and practical applications of this method across various domains of medical education. Hand gesture analogy teaching significantly enhances student learning by improving engagement, spatial reasoning, and procedural knowledge retention more effectively than conventional instructional methods. The integration of modern educational technologies and standardized implementation frameworks further amplifies its effectiveness. Despite challenges in standardization and faculty development, this method shows promising potential for transforming medical education by bridging the gap between theoretical knowledge and clinical practice. Evidence-based recommendations are provided for incorporating hand gesture analogy teaching into clinical training programs, supporting its adoption as an innovative pedagogical tool in contemporary medical education.

## Introduction and background

Medical education in the 21st century faces unprecedented challenges as the complexity of healthcare and the demand for knowledge continue to grow exponentially. Traditional didactic teaching methods, while foundational, increasingly reveal limitations in engaging students and cultivating the comprehensive skill set required for modern clinical practice [1]. Recent studies indicate that most medical students experience significant difficulties translating theoretical knowledge into practical clinical skills, underscoring the urgent need for more effective pedagogical approaches [2].

Hand gesture analogy teaching has emerged as a promising pedagogical tool, uniquely combining nonverbal communication with visual learning principles to bridge the gap between abstract medical concepts and their practical applications. By utilizing the human hand as a natural anatomical reference point, this approach offers medical students an accessible and intuitive framework for understanding complex physiological and anatomical relationships [3]. Evidence suggests that incorporating gesture-based learning enhances knowledge retention compared to traditional lecture-based instruction, particularly in areas requiring spatial understanding and procedural competency [4,5].

This review addresses three critical objectives in analyzing the effectiveness of hand gesture analogy teaching in medical education. First, it examines the theoretical foundations supporting this pedagogical approach, drawing from cognitive science, educational psychology, and medical education research. Second, it evaluates the practical applications and implementation strategies across various domains of clinical medical education, including anatomy, surgical training, and clinical skills development. Third, it provides a comprehensive analysis of the method's effectiveness through a critical examination of empirical evidence and comparative studies with traditional teaching approaches. Through this comprehensive analysis, we aim to contribute valuable insights into the ongoing evolution of medical education methodologies and offer evidence-based recommendations for implementing innovative teaching strategies in clinical training programs.

## Review

Theoretical foundation

Nonverbal Communication in Medical Education

Nonverbal communication (NVC) is a fundamental yet frequently underutilized component in medical education. Research indicates that the majority of information in human interaction is conveyed through nonverbal channels, including facial expressions, gestures, and vocal modulation. In clinical settings, effective NVC has been directly correlated with improved patient outcomes, increased satisfaction rates, and reduced malpractice claims [6]. Studies from leading medical institutions, such as Massachusetts General Hospital, demonstrate that structured assessment of nonverbal behaviors through frameworks like EMPATHY (Eye contact, Muscles of facial expression, Posture, Affect, Tone of voice, Hearing the whole patient, Your response) significantly enhances medical students' ability to recognize and respond to patients' emotional cues [7]. Furthermore, in chronic disease management, healthcare providers who demonstrate strong NVC skills increase patient adherence rates and improve health outcomes [8].

Cognitive Theory of Gesture-Based Learning

The cognitive foundation of gesture-based learning is rooted in contemporary neuroscience and educational psychology research. Functional magnetic resonance imaging (fMRI) studies have shown that gesture processing activates multiple neural networks simultaneously, engaging both motor and cognitive areas of the brain [9]. This multimodal activation enhances information encoding and retrieval by creating robust neural pathways. Meta-analyses showed that medical students who learned through gesture-based instruction improved their spatial reasoning skills and retention of procedural knowledge compared to traditional learning methods [10]. Gestures serve as physical anchors for abstract concepts, creating concrete representations that bridge the gap between theoretical understanding and practical application in medical education.

Educational Psychology Basis for Analogy Teaching

The psychological foundation of analogy teaching is grounded in learning theories emphasizing the importance of connecting new information to existing knowledge structures. This approach aligns with cognitive load theory, suggesting that learning is optimized when information efficiently utilizes working memory capacity [11]. In medical education, analogical reasoning has proven particularly effective in teaching complex anatomical and physiological concepts, with studies showing an improvement in concept retention when analogies are systematically incorporated into instruction [12]. Integrating hand gestures with analogical teaching creates a powerful pedagogical tool that leverages both physical and cognitive learning pathways, enhancing comprehension and long-term retention of medical knowledge [13].

By integrating these theoretical foundations, the hand gesture analogy teaching method leverages NVC, cognitive learning processes, and educational psychology to create a more effective and engaging learning environment [6]. This approach addresses current challenges in medical education and aligns with evolving trends that emphasize personalized learning, advanced technology integration, and innovative teaching strategies to prepare future physicians.

**Figure 1:**
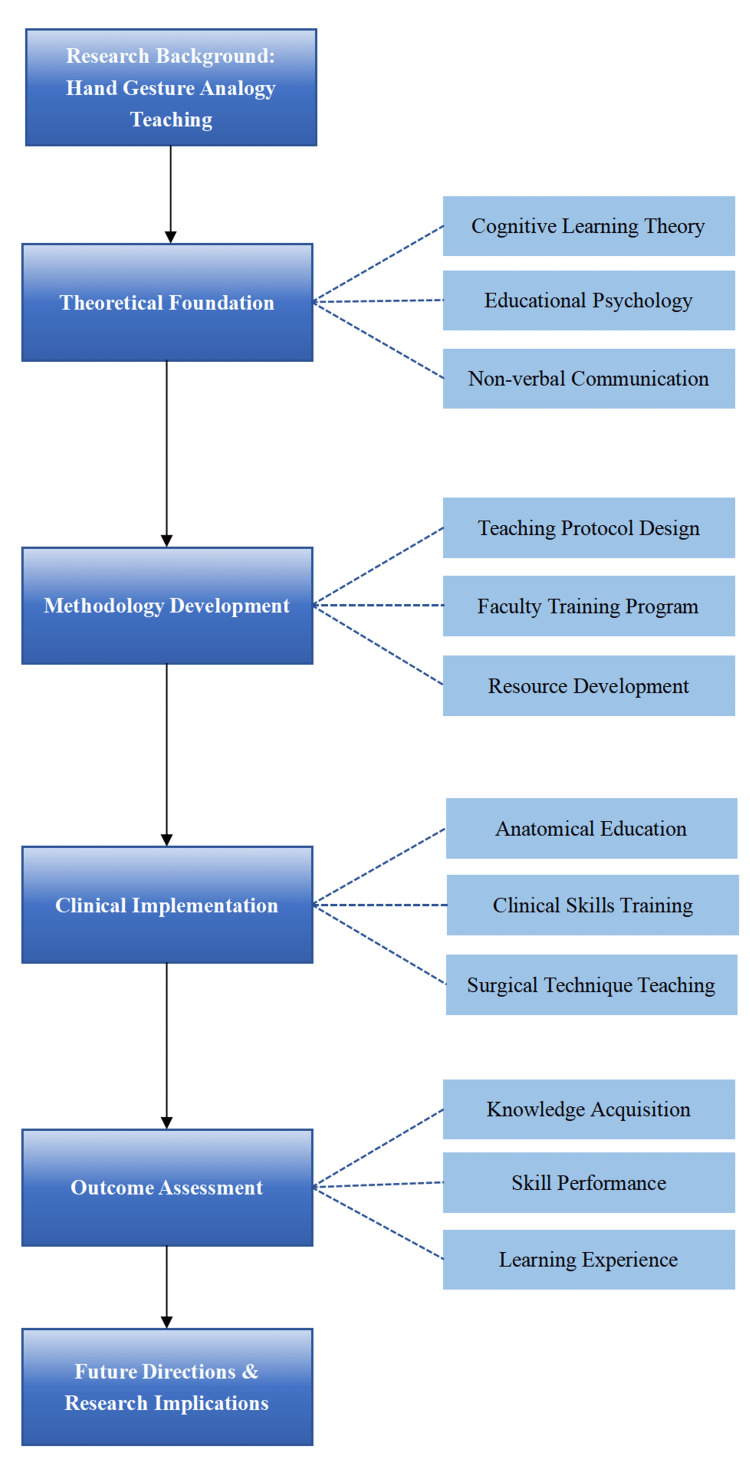
Conceptual framework of hand gesture analogy teaching in clinical medical education. The framework illustrates the systematic approach from theoretical foundation to practical implementation and assessment, highlighting the key components and their relationships in the teaching methodology. Image Credits: Guangbin Chen.

The conceptual framework (Figure 1) illustrates the systematic approach of hand gesture analogy teaching in clinical medical education. Beginning with the research background, which establishes the foundation and necessity of this teaching method, the framework progresses through several key phases. The theoretical foundation integrates cognitive learning theory, educational psychology, and non-verbal communication principles, providing the scientific basis for this approach. The methodology development phase encompasses teaching protocol design, faculty training programs, and resource development, ensuring systematic implementation. Clinical implementation focuses on practical applications in anatomical education, clinical skills training, and surgical technique teaching, demonstrating the method's versatility. The outcome assessment phase evaluates the effectiveness through knowledge acquisition, skill performance, and learning experience measurements. Finally, future directions address the ongoing development and potential improvements of this teaching methodology. 

Methodology development and implementation

Basic Principles of Hand Gesture Analogy Teaching

The hand gesture analogy teaching method is founded on principles of simplicity, accessibility, and interactivity. “Simplicity” refers to using straightforward gestures that are easily understood and replicated by students, ensuring that teaching aids do not overshadow educational content [14]. “Accessibility” emphasizes that the method can be employed in various settings, from traditional classrooms to virtual environments, without requiring sophisticated equipment [15]. “Interactivity” highlights engaging students through active participation, crucial for enhancing learning outcomes [14]. These principles guide the development of a teaching approach that is innovative and aligned with the educational needs of diverse student populations.

Teaching Design Framework

The teaching design framework for hand gesture analogy teaching involves several stages: needs assessment, instructional design, implementation, and evaluation. Needs assessment involves understanding the learning objectives and the prior knowledge of the students to tailor the gestures effectively [16]. Instructional design requires the creation of a curriculum map that integrates hand gestures with the content, ensuring that each gesture corresponds to a specific concept or structure [17]. Implementation involves the actual delivery of the curriculum, with instructors using gestures to illustrate complex ideas and facilitate student engagement. Evaluation entails assessing the effectiveness of the hand gesture analogy teaching method through various metrics, such as student performance, feedback, and engagement levels [18].

Integration With Modern Educational Technology

Integrating hand gesture analogy teaching with modern educational technology enhances its effectiveness and appeal. The use of 3D models allows students to visualize and interact with anatomical structures dynamically [19]. Combining this with hand gestures provides a multisensory learning experience catering to different learning styles. For example, when teaching the human skeletal system, an instructor can use hand gestures to mimic bone shapes and positions while referring to a 3D model for comprehensive understanding [19]. This integration aids in visualizing abstract concepts and promotes deeper cognitive engagement with the material [20].

Professional Development for Educators

Successful implementation of hand gesture analogy teaching requires comprehensive professional development for educators. This includes training on effectively using hand gestures to convey complex medical concepts and integrating these gestures with other teaching tools and technologies [21]. Educators must acquire the skills to adapt gesture analogies to different learning scenarios and assess their impact on student learning [22]. Professional development can take the form of workshops, seminars, and online courses, ensuring that educators are up-to-date with the latest research and best practices in hand gesture analogy teaching.

Standardization and Consistency

To maximize the benefits of hand gesture analogy teaching, standardization and consistency in the use of gestures across different educational settings are crucial [23]. This involves developing a set of standardized gestures for common medical concepts and terms, which can be adopted institution-wide or even nationally. Standardization ensures that students are not confused by disparate gestures used to represent the same concept in different classes or by different educators. It also facilitates the transfer of knowledge across different educational levels and institutions [24].

Scalability and Adaptability

The methodology must consider the scalability and adaptability of the hand gesture analogy teaching method as medical education evolves [25]. Scalability refers to the ability to expand the use of hand gestures to larger student cohorts and various medical specialties, while adaptability pertains to the flexibility to modify gestures based on feedback and evolving educational needs [26]. As medical education integrates new content, technologies, and pedagogical approaches, the hand gesture analogy teaching method should be able to accommodate these changes to remain effective and relevant.

By carefully developing and implementing the hand gesture analogy teaching method with these considerations, medical educators can provide a rich, interactive learning experience that enhances student engagement, understanding, and retention of complex medical concepts [27]. This method not only prepares students for clinical practice but also fosters a learning environment that is responsive to the changing demands of medical education.

Applications in clinical medical education

Anatomical Education

**Figure 2:**
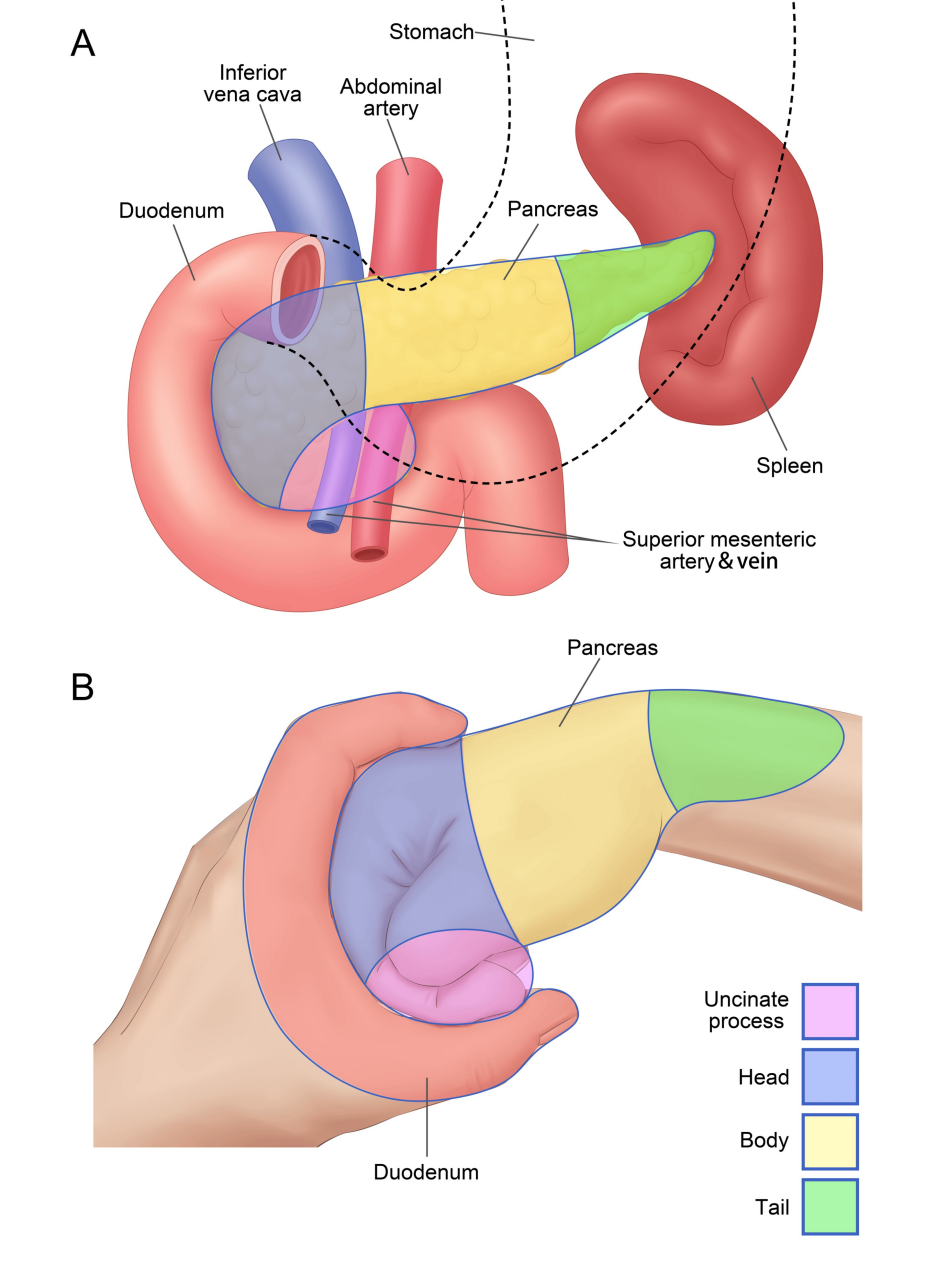
Hand gesture teaching method in pancreas anatomy. Using the hands to represent the pancreas and duodenum, with the left hand in a "C" shape and the right hand in a fist: the uncinate process is shown by the distal and middle phalanges of the right index finger and thumb tip (purple); the pancreatic head by the proximal phalanges of the right index finger and thumb base (blue); the body by the dorsal part of the right hand (yellow); and the tail by the right wrist (green). Original image credits by Guangbin Chen and published in the *Asian Journal of Surgery* [30].

Hand gesture analogy teaching has profound applications in anatomical education, a cornerstone of clinical medical training [24]. By leveraging the natural similarities between the human hand and various anatomical structures, this method provides a tangible and intuitive approach to understanding complex anatomical relationships [22]. For instance, the hepatobiliary system can be effectively illustrated by using the hand to represent the liver lobes and fingers to depict the bile ducts, making the intricate anatomy more accessible and memorable [28,29]. Similarly, pancreatic anatomy, with its head, body, and tail, can be analogized to parts of the hand (Figure 2), enhancing students' spatial understanding and recall [30]. The musculoskeletal system benefits from this method as well, with gestures facilitating the visualization of bone structures and joint movements, thereby reinforcing the connection between anatomical knowledge and clinical practice [31].

Clinical Skills Training

Surgical procedures: Hand gesture analogy teaching is particularly beneficial in surgical education, where precise hand-eye coordination and understanding of spatial relationships are crucial [32]. By mimicking surgical movements and instrument handling with hand gestures, students can develop a better sense of procedural steps and technical nuances before actual clinical exposure [33].

Physical examination: This teaching method can be used to demonstrate the techniques of physical examinations, such as auscultation or palpation, through gesture-based mimicry. This not only aids in learning technical skills but also in understanding the patient's perspective, enhancing empathy and communication skills in medical students [24].

Clinical diagnosis: In diagnosing clinical conditions, hand gestures can be employed to illustrate symptom presentations or pathophysiological mechanisms [20]. For example, using hand movements to depict the progression of a disease or the spread of infection can provide a dynamic and visual learning experience that complements didactic instruction [34].

Integration With Other Teaching Methods

3D visualization: The hand gesture analogy teaching method can be synergistically combined with 3D visualization technology. By correlating physical gestures with digital models, students gain a more comprehensive and interactive learning experience, which is particularly effective in understanding three-dimensional structures and spatial relationships in anatomy [35].

Clinical case studies: Integrating hand gestures with case-based learning allows students to connect theoretical knowledge with real-world clinical scenarios [36]. This approach fosters critical thinking and problem-solving skills, as students can physically "navigate" through a case using gestures, enhancing their understanding of disease mechanisms and management strategies [37].

Simulation training: Simulation-based medical education has been recognized for its potential to engage learners in behaviors similar to actual performance without real-world consequences. Hand gesture analogy teaching can augment simulation training by providing a bridge between the simulated environment and actual clinical practice [38], especially in procedures that require manual dexterity and spatial awareness [39].

The integration of hand gesture analogy teaching with these advanced educational strategies not only enhances the traditional learning experience but also aligns with current trends in medical education that emphasize collaboration, innovation, and diversity [9]. By embracing such innovative teaching methods, medical education can better prepare students for the dynamic and complex healthcare environment of the future, fostering a resilient and adaptable workforce ready to address modern healthcare challenges.

Assessment methods

Assessment methods are pivotal in evaluating the effectiveness of the hand gesture analogy teaching method [40]. The following methodologies are employed to gauge the impact on student learning.

Knowledge Tests

Standardized tests and quizzes assess the retention and understanding of medical concepts taught through hand gestures compared to traditional methods [26].

Practical Skills Evaluations

Objective Structured Clinical Examinations (OSCEs) and practical assessments measure the proficiency of clinical skills acquired through gesture-based teaching [41].

Student Feedback

Surveys and focus groups gather qualitative data on student perceptions, engagement levels, and satisfaction with the hand gesture analogy teaching method.

By employing these evaluation methods, a comprehensive understanding of the effectiveness of the hand gesture analogy teaching method in clinical medical education can be achieved. This data not only aids in refining the teaching method but also supports advocating for its wider adoption based on evidence of its benefits over traditional teaching approaches.

Challenges and solutions

Implementation Barriers

Integrating the hand gesture analogy teaching method into clinical medical education faces several implementation barriers. A significant challenge is the lack of infrastructure and technology, particularly in low- to medium-income countries [42]. Many institutions struggle with basic technological needs, such as reliable internet access and adequate digital tools, which can hinder the effective use of innovative teaching methods [42,43]. Additionally, poor communication within educational institutions can lead to fragmented implementation of new teaching strategies [44]. Without clear institutional support and direction, the adoption of innovative methods often fails to gain traction [45].

Faculty Development Needs

Successful implementation of the hand gesture analogy teaching method necessitates comprehensive faculty development. Educators must be trained not only in the mechanics of using gestures effectively but also in integrating these gestures with existing curricula and teaching technologies. Faculty members often require support in adapting their teaching styles to incorporate more interactive and student-centered approaches [46,47]. Training can take the form of workshops, mentorship programs, and collaborative learning opportunities that emphasize the importance of innovative teaching methods in enhancing student engagement and learning outcomes [48].

Standardization Issues

Another challenge is the standardization of gestures across different educational settings. The effectiveness of the hand gesture analogy teaching method relies on the consistent use of gestures to represent specific concepts. Without a standardized approach, students may become confused if different instructors use varying gestures for the same anatomical structures or clinical concepts [49]. Establishing a set of standardized gestures that can be universally adopted within medical education would help mitigate this issue and enhance the clarity of instruction [22].

Resource Requirements

Implementing the hand gesture analogy teaching method also requires adequate resources. This includes not only technological tools but also teaching materials and training programs for educators [50]. Ensuring that institutions have the necessary resources to support this innovative teaching method is crucial for its successful adoption and sustainability.

Development Recommendations

For the successful integration of hand gesture analogy teaching and other innovative methods into medical education, several recommendations can be made, which are discussed as follows.

Structured faculty development programs: Developing structured faculty development programs that focus on innovative teaching strategies, including the hand gesture analogy method, will equip educators with the skills needed to enhance their teaching practices [51]. These programs should emphasize active learning, student engagement, and the integration of technology [52].

Establishing standardized guidelines: Creating standardized guidelines for the use of hand gestures in medical education will provide clarity and consistency for both educators and students [53]. This can involve collaboration with educational experts to develop a comprehensive set of gestures that can be widely adopted across institutions.

By proactively addressing these challenges and implementing effective solutions, the hand gesture analogy teaching method can be successfully integrated into clinical medical education, ultimately enhancing student learning outcomes and preparing future healthcare professionals for the complexities of modern medicine.

## Conclusions

Hand gesture analogy teaching represents a significant innovation in medical education, offering a unique approach to enhance student learning and engagement. Its foundation in cognitive science and educational psychology, combined with practical applications across various medical education domains, positions it as a valuable complement to traditional teaching methods. While implementation challenges exist, including the need for standardization and faculty development, the method's potential benefits warrant continued investment and research. Future directions should focus on developing standardized protocols, enhancing faculty training programs, and investigating the method's effectiveness across different cultural and educational contexts. This teaching approach shows promise in preparing future healthcare professionals who can effectively bridge the gap between theoretical knowledge and clinical practice.
